# LiDAR Echo Gaussian Decomposition Algorithm for FPGA Implementation

**DOI:** 10.3390/s22124628

**Published:** 2022-06-19

**Authors:** Guoqing Zhou, Xiang Zhou, Jinlong Chen, Guoshuai Jia, Qiang Zhu

**Affiliations:** 1 School of Microelectronics, Tianjin University, Tianjin 300072, China; zqx0711@tju.edu.cn; 2 The Center of Remote Sensing, Tianjin University, Tianjin 300072, China; 3 Guangxi Key Laboratory of Spatial Information and Geomatics, Guilin University of Technology, Guilin 541004, China; 6614041@glut.edu.cn; 4 College of Mechanical and Control Engineering, Guilin University of Technology, Guilin 541004, China; 20200801035g@cqu.edu.cn (J.C.); 610912389@glut.edu.cn (G.J.); 5 College of Earth Science, Guilin University of Technology, Guilin 541004, China

**Keywords:** LiDAR, echo processing algorithm, Gaussian decomposition, FPGA

## Abstract

As the existing processing algorithms for LiDAR echo decomposition are time-consuming, this paper proposes an FPGA-based improved Gaussian full-waveform decomposition method. The proposed FPGA architecture consists of three modules: (i) a pre-processing module, which is used to pipeline data reading and Gaussian filtering, (ii) the inflection point coordinate solution module, applied to the second-order differential operation and to calculate inflection point coordinates, and (iii) the Gaussian component parameter solution and echo component positioning module, which is utilized to calculate the Gaussian component and echo time parameters. Finally, two LiDAR datasets, covering the Congo and Antarctic regions, are used to verify the accuracy and speed of the proposed method. The experimental results show that (i) the accuracy of the FPGA-based processing is equivalent to that of PC-based processing, and (ii) the processing speed of the FPGA-based processing is 292 times faster than that of PC-based processing.

## 1. Introduction

LiDAR (light detection and ranging) provides a significant means to acquire three-dimensional (3D) data [1]. LiDAR waveform analysis and decomposition is one of the most important research areas in LiDAR systems.

A lot of methods, considering different detection areas and the LiDAR operation environments, have been proposed over the past of decades. For example, Hofton et al. [2] proposed the Gaussian decomposition algorithm and evaluated the accuracy of the decomposition results using the root mean square error (RMSE). The key parameters of the Gaussian components include their amplitude, center position, and pulse width. Abdallah et al. [3] presented an underwater LiDAR waveform simulation model algorithm, which divides the returned waveforms in a water body into three parts: the water surface, the water body, and the water bottom. Cheng et al. [4] used the peak position of the GLAS (spaceborne earth science laser altimeter system) waveform to extract the relative height of ground targets and calculated the height of the building by identifying the peak position of the rooftop and the ground peak position in the waveform [5]. Gong et al. [6] used ICESat GLAS LiDAR data for urban environmental monitoring. In their study, the Gaussian decomposition method was used to estimate the height and density of buildings. Wang et al. [7] reviewed the methods involved in GLAS data processing and summarized some of the challenges faced, considering how to make better use of GLAS data. Brodu et al. [8] used 3D LiDAR to classify complex natural scene data under multi-scale criteria. Compared with the single-scale method, multi-scale analysis enhances the separability and spatial resolution of classification. Kinzel et al. [9] used the peak detection method in the RARRL software (which comes with the LiDAR system) in order to perform a second-order derivation of the echo waveform. Zhuang et al. [10] proposed an accurate and efficient ground peaking algorithm for a large “footprint” of LiDAR data. This algorithm sequentially combines a set of multi-scale second-order derivative filters and K-means clustering algorithms to avoid detecting “false” ground peaks. Pan et al. [11] used the wavelet decomposition algorithm to obtain the peak and position of the base reflectivity. Li et al. [12] proposed the use of small-footprint full-wave airborne LiDAR data to generate pseudo-large footprint waveforms for the hierarchical inversion of the orchard leaf area index. Liu et al. and Ma et al. [13,14] reviewed LiDAR echo processing algorithms and divided the existing LiDAR echo processing algorithms into mathematical simulation, deconvolution, and peak detection methods. Bruggisser et al. [15] proposed an algorithm that uses a tilted normal distribution function for waveform decomposition for tree species classification. Mountrakis et al. [16] proposed a linear approximate Gaussian decomposition algorithm, also known as the linear approximate iterative Gaussian decomposition (LAIGD) algorithm. Budei et al. [17] used Teledyne Optech’s multispectral airborne LiDAR (equipped with 1550 nm, 1064 nm, and 532 nm lasers) to identify tree genus or species. Song et al. [18,19] proposed a multi-spectral waveform decomposition method, which makes use of the principle that lasers of different wavelengths (556 nm, 670 nm, and 780 nm) have different reflectivity characteristics for the same target. Zhou et al. [20] proposed supercontinuum laser hyperspectral LiDAR, where the laser hyperspectral echo signals at 50 band covering 400~900 nm at a spectral resolution of 10 nm are reflected by the ground target.

With the development of detection technology, it is extremely urgent to quickly obtain the information contained in LiDAR echoes. In order to obtain information at near real-time, traditional methods have to handle the LiDAR waveform data on a personal computer (PC). Such traditional methods have the following shortcomings: (i) as a PC is a serial instruction system, the processing speed makes it difficult to meet the demand of time urgency, thus leaving the abundant echo resources underutilized, (ii) the LiDAR system collects a lot of data, which leads to large storage and post-processing, and (iii) the large amount of data generated in the process of LiDAR detection leads to post-processing, making it difficult to meet the needs of real-time detection. For the reasons above, this paper proposes an FPGA-based LiDAR echo Gaussian decomposition algorithm. The contributions of this paper are as follows:

(i) proposing a new LiDAR echo Gaussian decomposition algorithm, which utilizes a pair of the Gaussian inflection points and eliminates the “false” inflection points using a judgment condition;
(ii) paralleling the proposed algorithm with a FPGA hardware architecture;
(iii) validating the accuracy and timeliness of the proposed method using two LiDAR datasets covering the Congo and Antarctic regions, respectively.

## 2. Improved Gaussian Decomposition Algorithm

### 2.1. Pre-Processing

LiDAR waveforms are usually superimposed as the sum of all reflected signals of the detected target mixed with background noise [21,22], which usually contains a large amount of high-frequency noise. The random fluctuations of these noises greatly reduce the accuracy of the final fit. When obtaining the amplitude of the Gaussian component in the hardware structure, it is necessary to obtain the maximum value out of the inflection points of the original echo waveform. Thereby, it is necessary to obtain the first derivative of this segment of the waveform in order to estimate the amplitude. If the waveform contains a lot of noise, a few complicated comparison methods are required to obtain the amplitude, which undoubtedly increases the complexity of the hardware structure. Therefore, a LiDAR waveform should be pre-processed before the LiDAR waveform is decomposed, which includes noise estimation and Gaussian filtering. Through the two steps, the existence of a large number of “false” inflection points, which impact the solution of Gaussian component parameters, can be removed.

Gaussian filtering for smoothing the background noise and facilitating the subsequent processing of LiDAR waveforms is expressed [22] by

(1) $$f(x)=\frac{1}{\sqrt{2\pi }\delta }\exp(-\frac{x^{2}}{2\delta^{2}}),$$

where *x* represents the sampling time and δ represents the pulse width of the fitted transmit waveform. The larger the value of δ, the wider the frequency band of the Gaussian filter.

**Figure 1:**
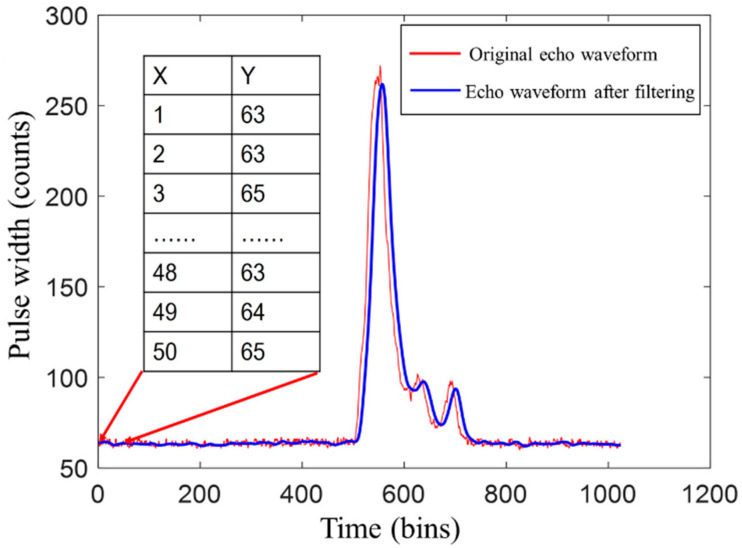
Comparison of LiDAR waveform and filtered waveform.

The calculation is carried out by using a specified template (or convolution mask) to scan each response strength in the echo waveform and replaces the response intensity value at the center of the template according to the response strength determined by the template. In this paper, the width of the Gaussian filter was set to 3 $\sigma_{t}$ and the length was set to 10 $\sigma_{t}$ (the high-speed AD sampling period), as shown in Figure 1.

When evaluating the noise of the full echo waveform, the beginning and end of the LiDAR echo waveform are both regarded as inactive and stable. The first 50 points of the echo signal are used to estimate the noise level, as shown in Figure 1. The area delimited by the two arrows represents the area used for evaluating the noise, where the first column of the table represents the time point and the second column represents the amplitude value corresponding to each time point, which is regarded as the background of the LiDAR waveform noise.

### 2.2. Inflection Point Coordinate Solution

According to the Gaussian decomposition algorithm [23] and the principle of the Gaussian function, two inflection points are obtained by taking the second-order derivative of the Gaussian function. The pulse width, half-wave width, and center position are directly dependent on the accuracy of the coordinates of the inflection points. The amplitude value depends on a certain value of the echo waveform between the inflection points. Therefore, it is necessary to obtain the exact inflection point coordinates before calculating the characteristic parameters (i.e., initial position, pulse width, half-wave width, and amplitude).

Previous research for solving the Gaussian inflection point has not specified the number of the inflection points [1,24]. This paper proposes pairing the Gaussian inflection points, where every fourth Gaussian inflection point is labelled as a Gaussian component. The conditions for finding the inflection points were refined using Equations (4) and (5). Before finding an inflection point, it was necessary to find the second-order difference of the LiDAR echo (i.e., to find the second-order derivative; see Figure 1). The process of obtaining the second-order difference from the Gaussian function is proposed by

(2) $$\Delta \left(\Delta y\left(x\right)\right)=y\left(x+2\right)+y\left(x\right)-2\times y\left(x+1\right).$$

where *x* is the time sample and *x* = 0, 1, …; *y*(*x*) is the amplitude sample at time *x*.

**Figure 2:**
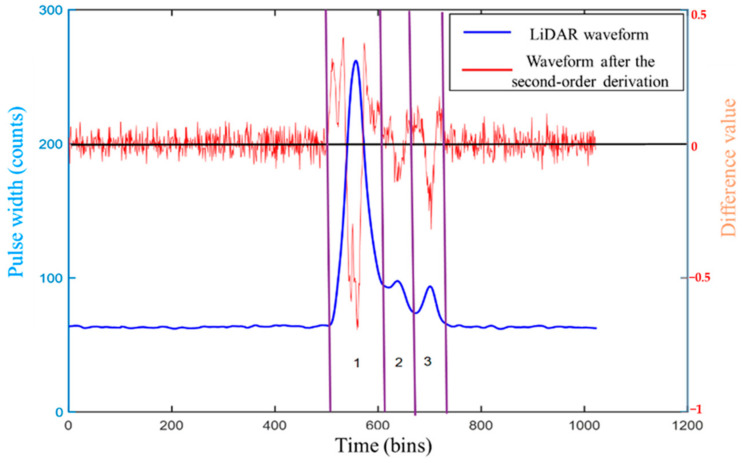
Comparison of the LiDAR waveform and the waveform after the second-order derivation.

The following situations may be encountered, as shown in Figure 2, when calculating the second difference using Equation (2).

In Figure 2, the blue line represents the LiDAR waveform, which can be decomposed into three Gaussian components which are the parts 1, 2 and 3, while the red line represents the waveform after the second-order derivation. After putting the LiDAR waveform and the second-order differential waveform into a diagram to find the second-order difference, it can be more significantly observed that each Gaussian component corresponds to a downward “spike”. The intersection of the second-order difference of the waveform and the *x*-axis is the inflection point of the Gaussian component and each LiDAR echo component corresponds to two inflection points, indicating that two adjacent odd–even inflection points, i.e., $x_{2i-1}$ and $x_{2i}$, can determine a Gaussian component.

**Figure 3:**
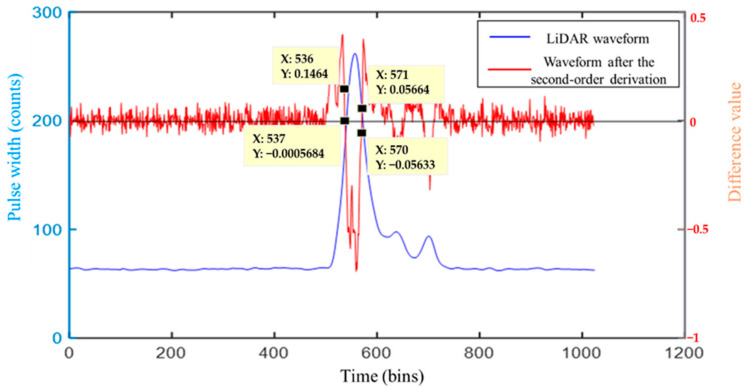
Inflection points near coordinates representation.

When the left and right inflection points of the Gaussian component are zero, the two inflection points are obtained. When the left and the right inflection points of the Gaussian component are zero, the two inflection points are obtained. However, sampling of the LiDAR echo results in the time-series, which is always discrete and, so, it is not always zero. Therefore, when calculating the inflection point of the LiDAR echo (the step length of *x* is set to 1), the inflection point on the left, $x_{2i-1}$, is determined by the adjacent coordinates, $x_{2i-1,left},y_{2i-1,left}$ and $x_{2i-1,right},y_{2i-1,right}$, of the upper and the lower sample points, which after second-order differencing are closest to the *x*-axis, while $x_{2i-1}$ matches the nearest inflection point, $x_{2i}$, which needs the following to be satisfied: $y_{2i-1,left}$ < 0 and $y_{2i-1,right}$ < 0. Then, $x_{2i-1}$ and $x_{2i}$ are used to determine an undetermined Gaussian component. A total of four adjacent coordinates of the sample point are required to determine a Gaussian component. Therefore, the judgment of an undetermined Gaussian component is given by (see Figure 3):

(3) $$y_{weight\_i}=\left\{\begin{array}{l} 1\;\;if\{(y_{2i-1}=0)||(y_{2i-1,left}>0\;\&\;\&\;y_{2i-1,right}>0)\}\;\&\;\&\\ \;\;\;\;\{(y_{2i}=0)||(y_{2i,left}<0\;\&\;\&\;y_{2i,right}>0)\}\\ 0\;\;\mathrm{else} \end{array}\right.,$$

where $y_{weight\_i}$ represents the undetermined Gaussian component and $x_{2i-1}$ and $x_{2i}$ represent the left and right inflection points of the undetermined Gaussian component, respectively.

The adjacent coordinates of the two inflection points corresponding to the Gaussian component of the LiDAR waveforms are (536, 0.1464), (537, −0.0005684), (570, −0.05633), and (571, 0.05664), respectively (see Figure 3). As each pair of Gaussian inflection points requires four proximity coordinates, we substituted the above four coordinates into Equations (4) and (5) to obtain the specific locations of the left inflection point, *x*_2*i*−1_, and the right inflection point, *x*_2*i*_.

(4) $$x_{2i-1}=x_{2i-1,left}+\frac{y_{2i-1,left}}{y_{2i-1,left}+|y_{2i-1,\mathrm{right}}|},$$

(5) $$x_{2i}=x_{2i,left}+\frac{y_{2i,\mathrm{right}}}{y_{2i,\mathrm{right}}+|y_{2i,left}|},$$

where $x_{2i-1,\mathrm{left}},y_{2i-1,\mathrm{left}}$ and $x_{2i-1,\mathrm{right}},y_{2i-1,\mathrm{right}}$ represent the adjacent coordinates of the two inflection points.

### 2.3. Gaussian Component Parameter Solution

**Figure 4:**
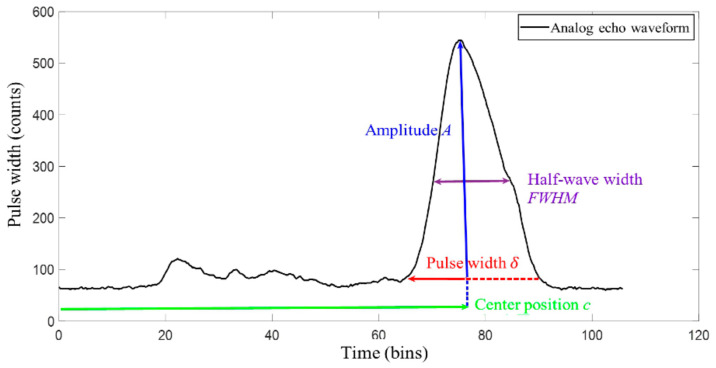
Analog echo waveform.

The parameters (such as amplitude, center position, pulse width, and half wave width) are implicit in a single Gaussian waveform (see Figure 4). According to the inflection point coordinate solution method described in Section 2.2, the left and right inflection points, $x_{2i-1}$ and $x_{2i}$ of the undetermined Gaussian component can be obtained, respectively. The estimated value of the center position $c_{i}$ and the pulse width $\delta_{i}$ of each Gaussian component are solved using the positions and intervals of consecutive inflection points. The relationship for the center position $c_{i}$ and pulse width $\delta_{i}$ are calculated using Equations (6) and (7), respectively [25]. The position and intensity (*x_max_*, *y_max_*) of the crest point, the corresponding center position *c_i_* and amplitude *a_i_* can be determined using Equation (8).

(6) $$c_{i}=\left(x_{2i-1}+x_{2i}\right)/2,$$

(7) $$\delta_{i}=\left|x_{2i-1}-x_{2i}\right|/n,$$

(8) $$a_{i}=y_{max}-\mu_{noise},$$

where *x*_2i−1_ and *x*_2*i*_ represent a set of inflection points corresponding to the Gaussian component, respectively; *n* represents the undetermined coefficient; $y_{\max}$ represents the maximum amplitude; and $\mu_{noise}$ represents the average background noise.

### 2.4. Echo Component Location

As different reflectors have different reflectivities in response to the laser, meanwhile, the backscattering of the LiDAR occurs, which further interferes with the determination the parameters of the LiDAR waveform. Simulation investigation of the LiDAR echo signal based the models and the actual echo signal shown in Figure 4, including the echo waveform and corresponding key parameters, i.e., the amplitude $\left(a_{i}\right)$, center position (*c_i_*), pulse width (*δ_i_*), and half-wave width (FWHM), was conducted. It can be observed, from Figure 4 that the LiDAR waveform was not completely symmetrical from left to right, like a high-beam waveform, where the left half of the echo component was more accurate, but the right half of the waveform had a large deformation.

Traditional methods, such as the centroid method, determine the echo time as the time corresponding to the local amplitude point; that is, the time point of the center position [26,27]. Therefore, the echo moment is determined by calculating the average of the center position and the transverse coordinates of the half-wave width *FWHM* and the intersection of the waveform on the left using Equation (9). In turn, the effects of using the right half-echo waveform component and the errors caused by a single reliance on the amplitude corresponding to the moment of the location can be avoided.

(9) $$t_{i}=t_{\max}-0.25t_{FWHM},$$

where $t_{i}$ represents the distance measurement point corresponding to the Gaussian component, $t_{max}$ represents the center position, and $t_{FWHM}$ represents the length of the half-wave width.

## 3. FPGA Implementation for the Improved Gaussian Decomposition Algorithm

### 3.1. FPGA Overall Hardware Architecture

**Figure 5:**
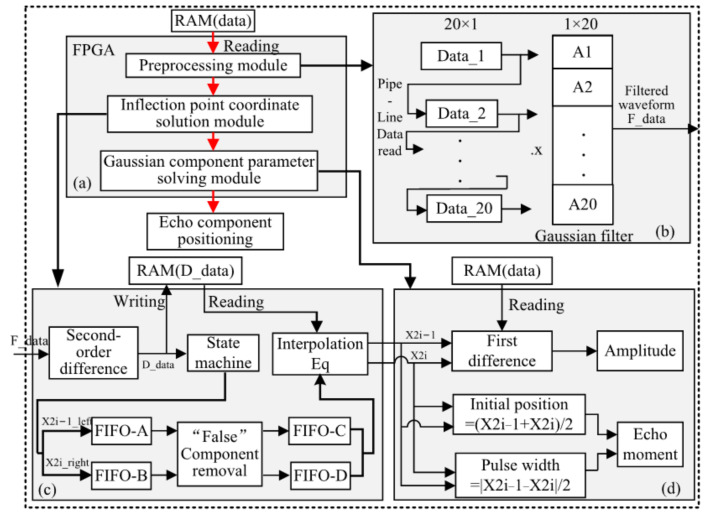
FPGA architecture: (**a**) overall architecture; (**b**) pre-processing architecture; (**c**) inflection point coordinate solution architecture; (**d**) Gaussian component parameter solution and echo component positioning architecture.

In order to fast the calculation for the proposed algorithm above, an FPAG hardware architecture is established (Figure 5a), mainly consisting of three modules, namely, the pre-processing module, the inflection point coordinate solution module, and the Gaussian component parameter solution and echo component positioning module. The pre-processing module (see Figure 5b) includes the data reading and Gaussian filtering pipeline. After reading the waveform of the LiDAR echo from RAM, the waveform enters the FPGA through the pipeline, and the filtered waveform was obtained after the Gaussian filtering convolution operation. The second module is for inflection point coordinate solution (see Figure 5c). The data is divided into two channels after the second-order differential operation—one is stored in RAM, while the other enters the state machine for detection. The coordinates that meet the requirements are stored in FIFO-A and FIFO-B and interpolation is used to calculate the inflection point coordinates. The third module involves the solution of the Gaussian component parameters and echo component positioning (see Figure 5d). The Gaussian component and echo time parameters are obtained through this step. The details of FPGA-based implementation for each module are described in the following sections.

### 3.2. Submodule

#### 3.2.1. Pre-Processing Module

The pre-processing module includes a RAM data reading module and a Gaussian filter. The RAM data are read in a pipeline structure, according to the parallel characteristics of the FPGA. The structure of the Gaussian filter also needs to meet the requirements of parallelism.

A. RAM data reading module

**Figure 6:**
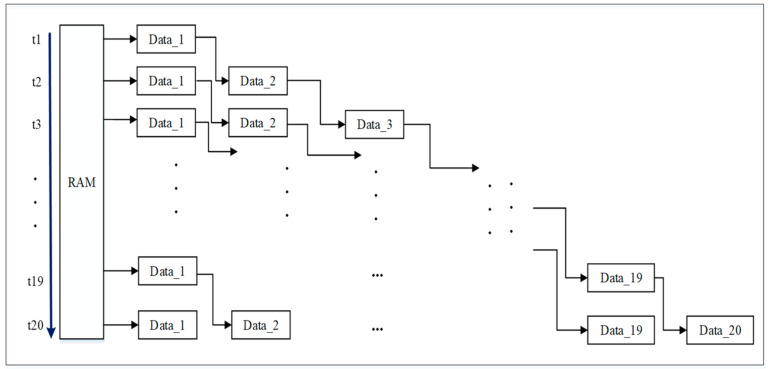
RAM data read module.

Pipeline processing is a commonly used design method for FPGAs [28,29]. In order to meet the requirements of the subsequent filtering. The reading module is designed using a pipeline mode. The initial waveform of the LiDAR echo is stored in a RAM IP with a width of 11 bits and a depth of 528/1024. The architecture for FPGA pipeline data reading is shown in Figure 6.

As shown in Figure 6, the waveform values of the LiDAR echo are stored in RAM, denoted as $y_{i}=\left\{y_{1},y_{2},\ldots ,y_{n}\right\}$. There are 20 variables, data*_i_* (*I* = 1, …, 20). In the first cycle, t_1_, *y*_1_ is read from RAM and assigned to data_1_. In the second cycle, t_2_, *y*_2_ is read from RAM and assigned to data_1_; at the same time, the value *y*_1_ of data_1_ in the previous cycle is assigned to data_2_. By the twentieth cycle, t_20_, the value of data_20_ is assigned *y*_1_, the value of data_19_ is assigned *y*_2_, and so on, until the value of data_2_ is *y*_19_ and the value of data_1_ is *y*_20_. Data_1_ to data_20_ begun being output at the twenty-first period t_21_, where the output data from data_1_ to data_20_ are $y_{20}$ to $y_{1}$. In the 22nd cycle, the output data from data_1_ to data_20_ are *y*_21_ to *y*_2_, and so on. The pipeline data reading module in each cycle simultaneously outputs twenty values to the Gaussian filter module.

B. Gaussian filter module

The filter pulse response Equation (1) contains a lot of decimals and involves a lot of floating-point operations, which result in difficulties for FPGA-based implementation, i.e., it consumes a lot of DSP arithmetic units [29,30]. Therefore, this paper transforms it into shift, addition, and multiplication operations using the conversion shown in Equations (10) and (11).

h = [0.0210, 0.0269, 0.0336, 0.0409, 0.0483, 0.0555, 0.0620, 0.0674, 0.0712, 0.0732, 0.0732, 0.0712, 0.0674, 0.0620, 0.0555, 0.0483, 0.0409, 0.0336, 0.0269, 0.0210].(10)

After normalizing and rounding the above matrix, we have

(11) $$A=1/2^{11}[43,55,69,84,99,114,127,138,146,150,150,146,138,127,114,99,84,69,55,43].$$

**Figure 7:**
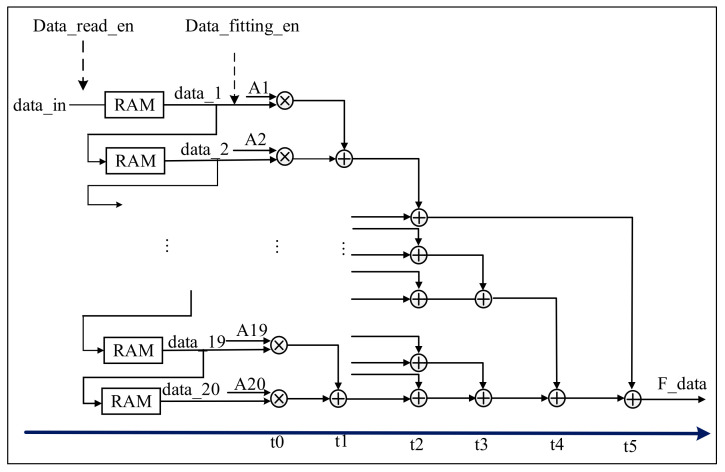
Filter module.

If the above data are directly shifted, the 11th bit of data will be lost. However, in order to save the consumption of resources, ensure the accuracy requirements of the algorithm, and balance the consumption of resources, a floating-point division IP core is added at the end of the filter. The hardware structure is shown in Figure 7.

The 20 integer multiplication IP cores and the 19 integer adder IP cores are used in the filter hardware structure (see Figure 7). Starting from the 21st cycle, when data_fitting_en is high, the variables data_1_ to data_20_ of the pipeline data reading module output 20 data. The data output in RAM is multiplied by the 20 data from A1 to A20, respectively. The design of the t_0_ column uses 20 integer multiplier IP cores, and the output is 20 data. The design of the t_1_ column uses 10 integer adder IP cores, and so on, the t_2_ column uses five integer adder IP cores. The t_3_ column uses two integer adder IP cores. At this time, there is one datum left, and two data are output, or there are three data remaining, and two additions are needed. The t_4_ column uses an integer adder IP core, which outputs one datum. The t_5_ column uses an integer adder IP core, adds the output data in the t_4_ column and the remaining data in the t_2_ column, and then outputs one datum. Finally, a 64-bit floating-point multiplication IP core is used to reduce the data by 2^11^, to form the output. However, the second-order difference operation is required after filtering. The second-order difference operations mainly include addition and subtraction, and the floating-point IP core is good at processing multiplication/division operations, while consuming a lot of resources when performing addition/subtraction operations. Therefore, we transferred the double-precision floating-point division IP core that should have been added after the Gaussian filter module to the last step of the second-order difference module; that is, the double-precision floating-point IP core was added to the inflection point coordinate solution sub-module.

#### 3.2.2. Inflection Point Coordinate Solution Module

The inflection point coordinate solution module consists of a second-order difference module, an inflection point coordinate query module, and an inflection point coordinate calculation [31,32,33].

A. Second-order difference module

**Figure 8:**
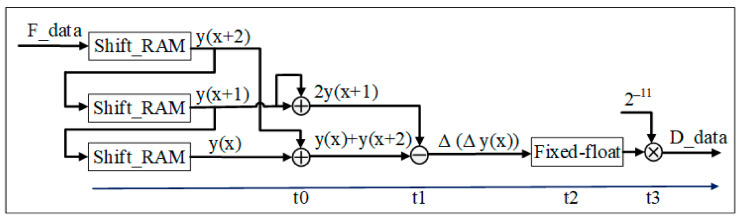
Second-order differential module.

Two additions, one multiplication, and one subtraction in the second-order difference module are involved. The architecture of the second-order difference operation is shown in Figure 8. This module uses a total of 7 IP cores, including two 24-bit shift register IP cores, two 24-bit integer addition register IP cores, a 25-bit integer subtraction register IP core, and a 24-bit integer to floating-point IP core, respectively. The output data, D_data_, is 64-bit floating point.

When the filtered data, F_data, is input, the second-order differential module and the RAM data read module have the same pipeline read working mode. After two cycles of buffering, three shift registers output at the same time; as shown in Equation (2), the data of shift_RAM2 (*y*(*x* + 1)) plus itself constitutes the next subtraction. The data *y*(*x* + 2) of shift_RAM1 plus the data *y*(*x*) of shift_RAM3 constitute the next subtracted number. As integers cannot be directly input to the double-precision floating-point division IP core, this method first uses an integer-to-double-precision floating-point IP core, based on the previous step, to convert integer data into double-precision floating-point; then, double-precision floating-point division is performed; finally, the 64-bit double-precision floating-point data are output to the next module.

B. Inflection point coordinate query module

**Figure 9:**
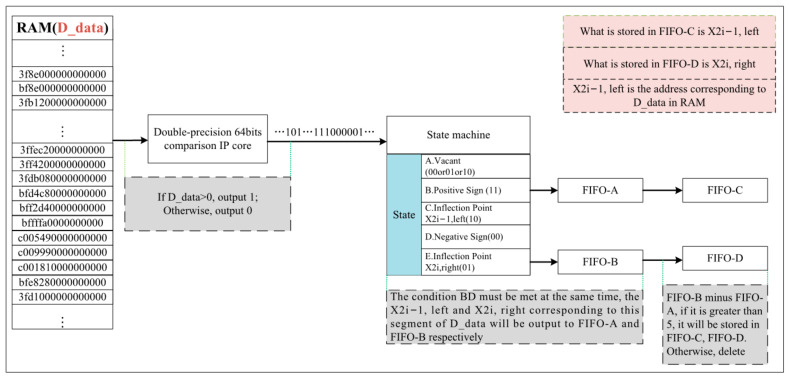
Inflection point coordinate query module.

**Figure 10:**
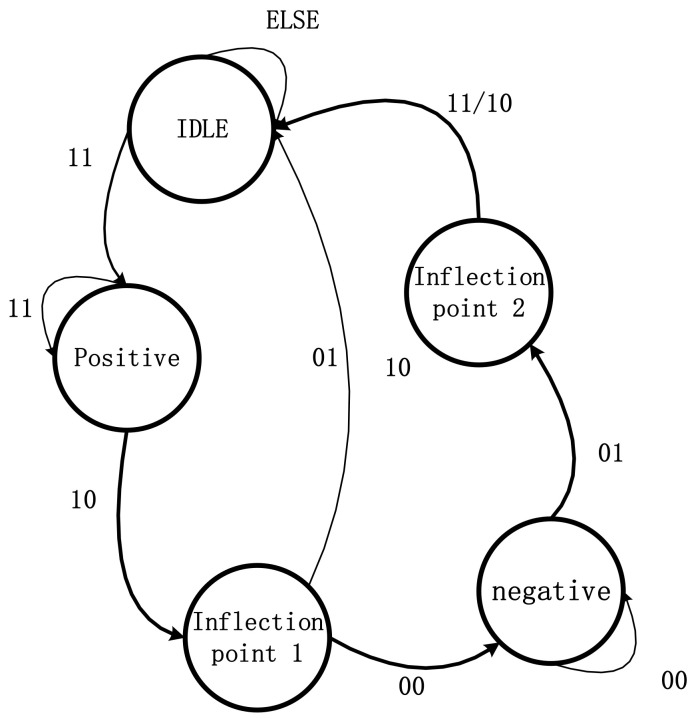
Schematic diagram of state machine.

The output data, D_data, in Figure 8 are the 64-bit double-precision floating-point data after the second-order difference operation, which are stored in RAM as the input in Figure 9. Then, D_data is input into the floating-point comparison IP core in order to compare the size of the input and zero. When the data is greater than 0, the output is 1; when the data is less than 0, the output is 0. In general, the data after the second-order difference operation *ddy,* corresponding to a set of inflection point coordinates *x*, is in the form of …11100…1… Furthermore, the module designs a state machine (the specific structure of the state machine is shown in Figure 10) to store all pairs of adjacent coordinates (10, 01) of the inflection point in the RAM, which conform to Equation (3) into the FIFO.

When the value of 10 is detected, the enable state of FIFO_A (FIFO_A_wr_en) is “1”, and the coordinate $x_{2i-1,\mathrm{left}}$ corresponding to 10 is stored in FIFO_A. Then, when the value of 01 is detected, the enable state of FIFO_B (FIFO_B_wr_en)) is “1”, and the coordinate $x_{2i-1,\mathrm{right}}$ corresponding to 01 is stored in FIFO_B. Next, we use the value of FIFO_B is used to subtract the value in the corresponding FIFO_A. If the value is less than 2, the echo component, whose pulse width is less than 4 ns, is removed. If the value is greater than 5, the values are shifted from FIFO_A and FIFO_B to FIFO_C and FIFO_D. The output is a pair of adjacent coordinates corresponding to the inflection points $x_{2i-1}$ and $x_{2i}$.

C. State machine

The state machine has five states; namely, IDLE, positive, inflection point 1, negative, and inflection point 2. Each state must detect the positive and negative states of two consecutive points. Assuming a continuous sequence {1, 2, 3, 4, 5, …}, the first detection is 1 and 2, the second detection is 2 and 3, and so on. First, when the initial state is IDLE, check whether the state of the data meets the state of 11; if not, the state is IDLE, and continue to check whether the state of the next group of data meets the state of 11. If the status is 11, then jump to the next state: positive. When the current state is positive, the next group sequence is detected, if the next group of data status is 11, the state is positive; otherwise, the state is 10 is detected, and we jump to the next state: inflection point 1. When the current state is inflection point 1, check the next set of data. If the next set of data state is 01, as the interval between inflection points 1 and 2 is too small, it is judged that this set of inflection points are a “False turning point,” and the next state jumps to IDLE. If the state of the next group of data is 00, then jump to the next state: negative. If the state of the next group of data is 01, the next state is inflection point 2; if the current state is inflection point 2, no matter whether the state of the next group of data is 11/10, the next state will be IDLE. Then, the group of obtained inflection points are judged. After judging a set of inflection points, store the coordinates of inflection point 1 in FIFO_A and store the corresponding inflection point 2 in FIFO_B.

D. Inflection point coordinate calculation module

**Figure 11:**
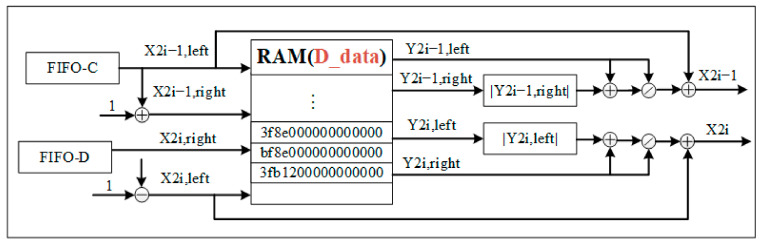
Inflection point coordinate calculation module.

As shown in Figure 11, the inflection point coordinates, *x*_2i−1_ and *x*_2*i*_, are calculated using Equations (4) to (5). The abscissa of the coordinate near the inflection point is input into RAM (D_data_), and the corresponding ordinate is output. Then, the absolute value, addition, division, and addition operations are performed in parallel mode in order to obtain a set of inflection points corresponding to Gaussian components.

#### 3.2.3. Gaussian Component Parameter Solving and Echo Component Positioning Module

After calculating the inflection point, two sub-modules, a key parameter solving module (with respect to the amplitude, *a_i_*, the center position, $c_{i}$, and the pulse width, $\delta_{i}$) and the result output module, are designed. The details are described below.

A. Solving the amplitude *a_i_*

**Figure 12:**
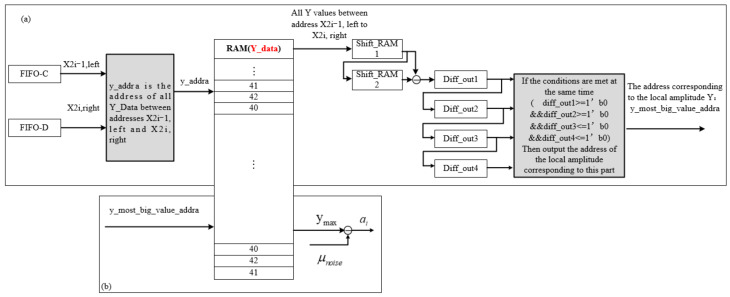
The calculation for the amplitude of each Gaussian component: (**a**) Solving for the maximum value, *y_max_*; (**b**) Solving for the amplitude $a_{i}$.

The calculation for the amplitude corresponding to each Gaussian component is shown in Figure 12. First, the adjacent coordinates of the inflection point calculated in the previous step are input into the module, and the original echo waveform is stored in RAM. Then, part of the waveform, are according to the adjacent coordinates of the inflection point, and set the read address of RAM to yaddra, such that the value range of yaddra is between the two adjacent coordinates of the inflection point [32,33].

After extracting the data between the two inflection points, we extract the local maximum, *y_max_*, is extracted from it. The intercepted part of the waveform is subjected to the first-order difference operation, through shift_RAM. We compare the output results each time. According to the principle of local maximum, the first half of the Gaussian waveform is increasing, and the second half is decreasing, so the result of the difference is …111000… If the condition 1100 is met, it is judged to be the address corresponding to the maximum value, *y_max_*, of this Gaussian component. Then, we input its address into RAM (Y_data_) and output the maximum value of the Gaussian component *y_max_*. Finally, the amplitude, $a_{i}$, is calculated using Equation (8).

B. Solving the center position *c_i_*, pulse width *δ_i_*, and echo component positioning module

**Figure 13:**
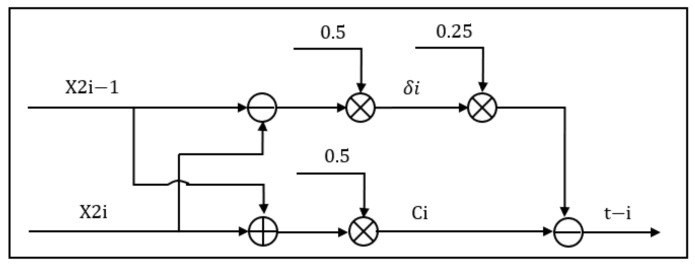
Solving center position $c_{i}$, pulse width $\delta_{i}$, and result output module.

With Equations (6) and (7), the module performs subtraction, addition, and multiplication operations on the inflection points corresponding to a group of Gaussian components in order to obtain the center position $c_{i}$ and pulse width $\delta_{i}$ of the Gaussian component. Then, it performs multiplication and subtraction to obtain the echo time, *t_i_*, as shown in Figure 13.

## 4. Experiments and Analysis

### 4.1. Data Sets

**Figure A1:**
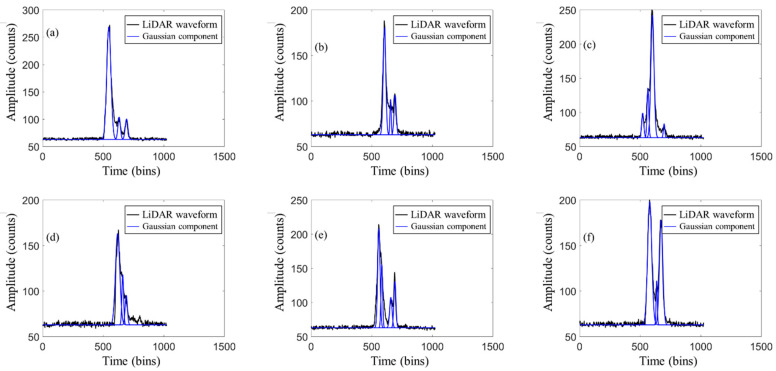

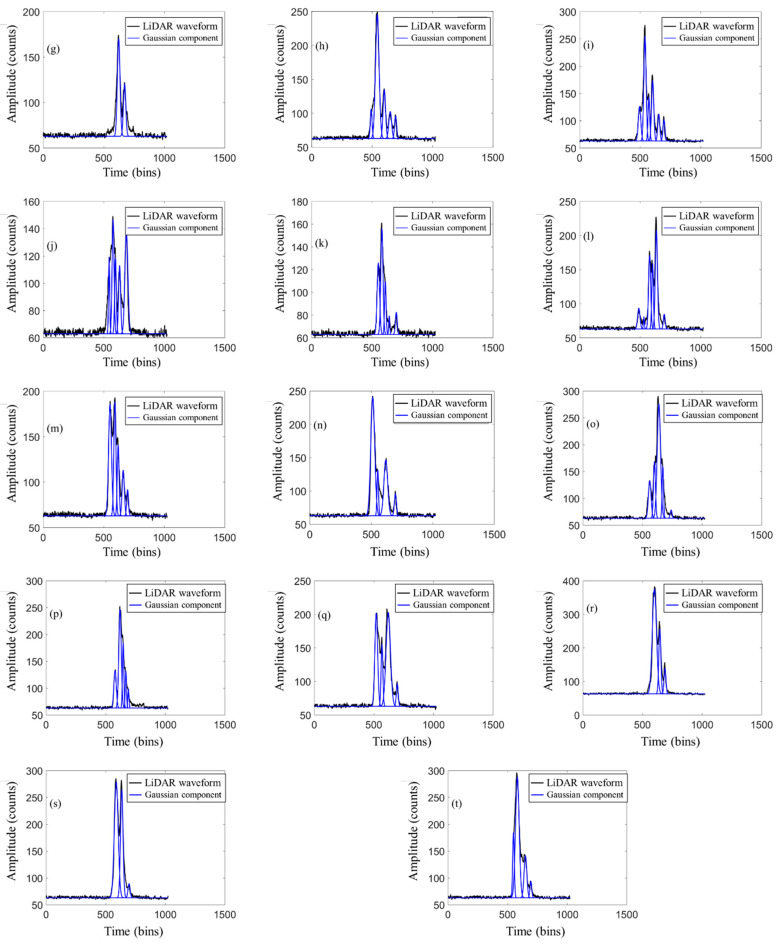
(**a**–**t**) LiDAR waveform data and LiDAR echo decomposition waveform in the Congo region. The abscissa is the sampling time point of the LiDAR waveform and the ordinate is the amplitude coordinate of the waveform.

**Figure A2:**
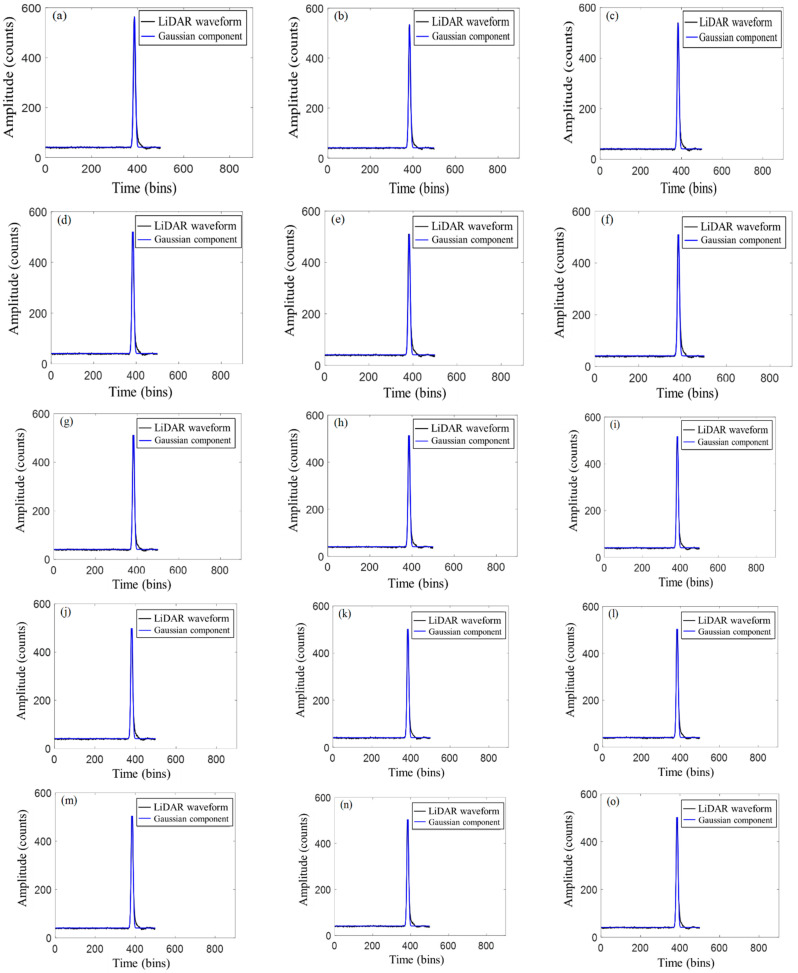

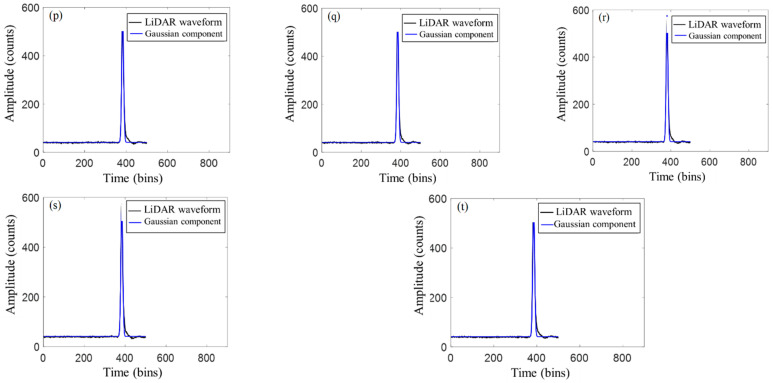
(**a**–**t**) LiDAR waveform data and decomposed waveforms of ocean LiDAR waveforms in Antarctica. The abscissa is the sampling time point of the LiDAR waveform and the ordinate is the amplitude coordinate of the waveform.

In order to verify the validity of the methods in this paper, experiments were conducted using data from NASA’s airborne full-waveform LiDAR system (LVIS), where the study areas are:

(1) The Congo. The experimental data were collected from February to March 2016, and the flight area was Gabon, Africa. A Langley King Air B-200 aircraft was fitted with an LVIS installation for data collection, flying at an average ground elevation of 24 km. The nominal LVIS strip width was 1.5 km (200 mrad), and the nominal LVIS footprint diameter was 18 m (2.5 mrad). There are a total of 400,000 sets of data in each set, and the size of each data set is 1 × 1024.
(2) Antarctica. The experiment was carried out in the Antarctic region is 2011. The LVIS was installed on NCAR’s G-V aircraft with an average ground altitude of 45 km. The nominal LVIS strip width was 2.7 km (200 mrad), and the nominal LVIS footprint diameter was 20 m (2.5 mrad). There are 700,000 sets of data in each set, and the size of each data set is 1 × 528.
(3) Appendix A Figure A1 black waveform shows 20 sets of echo waveform data in the land area of Congo, while Appendix B Figure A2 black waveform shows 20 sets of echo waveform data in the ocean area of Antarctica. For the experiment, the echo waveform of the measurement area was decomposed and located, which is mainly divided into two categories: land and ocean. Vivado was used to test these 40 groups of data in order to realize the real-time reliability and accuracy of the algorithm.
(4) In Appendix A Figure A1 black waveform, the abscissa is the sampling time point and the ordinate is the amplitude of the waveform. This paper includes as many various complex LiDAR echo waveforms as possible with different terrains, and the number of Gaussian components of each LiDAR echo ranged from 3 to 6.
(5) In Appendix B Figure A2 black waveform, the abscissa is the sampling time point and the ordinate is the amplitude of the waveform. Due to the small influence factors, such as wind and waves, in the ocean area, the echo waveforms are simpler and have fewer Gaussian components.

### 4.2. Echo Waveform Decomposition

The above forty groups of LiDAR waveforms were input into Vivado for simulation in order to realize the decomposition of LiDAR waveforms. After fitting the parameters of the Gaussian component, it was compared with the LiDAR waveform. It basically coincided with the LiDAR waveform. The fitted waveforms of the two sets of experiments are shown in Appendix A, Figure A1, and Appendix B, Figure A2, respectively.

In Appendix A, Figure A1(a,b), the blue waveform shows only three Gaussian components in each group of echo waveforms, and there are fewer echo components. Some data had more echo components, such as those of the blue waveform in Appendix A, Figure A1(h,i), where each group of echo waveforms contains five and six Gaussian components, respectively.

**Figure 14:**
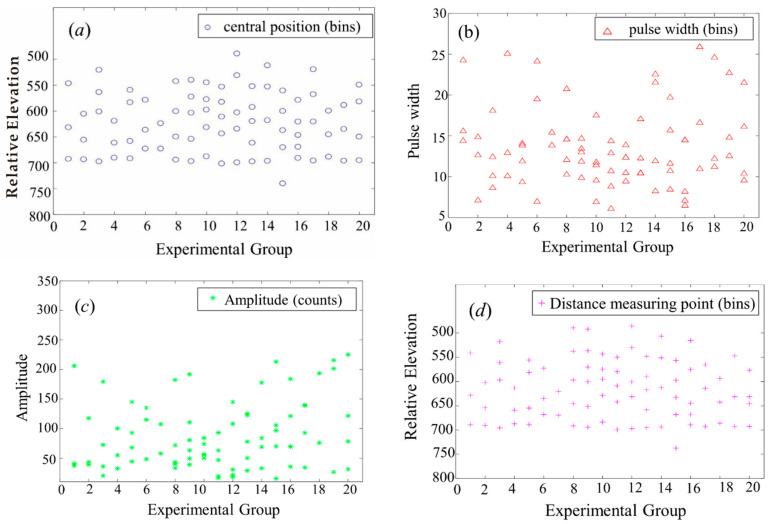
LiDAR echo decomposition results in Congo: (**a**) Central position; (**b**) Pulse width; (**c**) Amplitude; (**d**) Distance measuring point.

Figure 14 presents the key parameters of LiDAR echo decomposition results in Congo. The center position in Figure 14a and the content of the ranging point response in Figure 14d are all relative elevations. In order to display the relative elevation data, we reversed the y-coordinate direction of these two graphs. As the detection target is a tropical rain forest area, the relative elevation of the two images can reflect the information of the detected object from different angles. These Gaussian components may represent obviously different levels of the canopy, underlying layer, and ground. The last Gaussian component of the echo waveform is the farthest from the transmitted waveform, which represents the ground. The pulse width in Figure 14b is related to the target surface roughness, mostly concentrated between 7 and 16. The amplitude reflects the backscattering characteristics of the target, where the main value range is less than 150. Comparing Figure 14b,c, it can be seen that the physical properties of the target were basically the same.

The detection area for Antarctica, as shown in Appendix B, Figure A2, was mainly ocean and, so, the waveforms were relatively simple. The echo component was less prominent. Compared with Appendix A, Figure A1, the pulse width of the echo waveform is narrower, and the decomposition coefficient needs to be adjusted appropriately.

**Figure 15:**
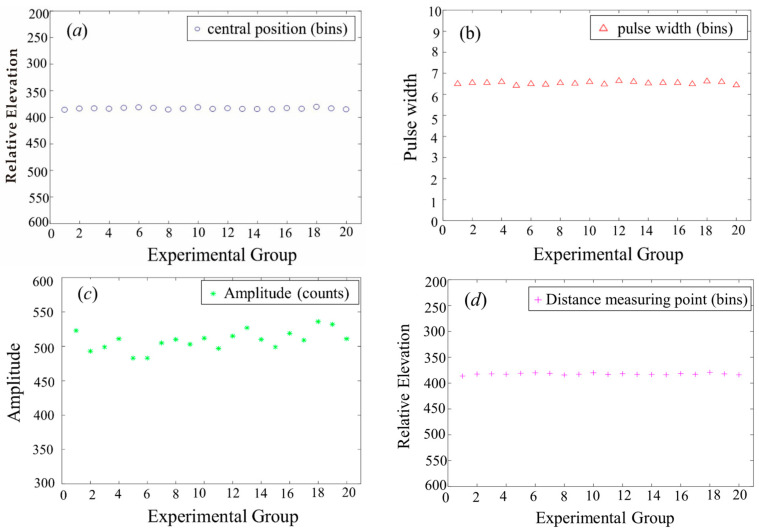
LiDAR echo decomposition results in Antarctica: (**a**) Central position; (**b**) Pulse width; (**c**) Amplitude; (**d**) Distance measuring point.

Detection targets on the sea surface are simple. The center position in Figure 15a and the content of the ranging point in Figure 15d are all relative elevations. The first Gaussian component of the echo waveform represents the sea surface. The y-coordinate direction of these two plots was reversed. The relative elevation of the two images represents the shape of the sea surface. The pulse width in Figure 15b is related to the surface roughness of the target. The amplitude of Figure 15c reflects the backscattering characteristics of the target. The values between the two figures tended to the same interval, indicating that the physical properties of the target were basically the same.

### 4.3. Error Analysis of Echo Waveform Decomposition

For waveform decomposition algorithms, the most commonly used error analysis standard is the root mean square error (RMSE) analysis method, as shown in Equation (12) [25]. We used the RMSE to make a certain comparison between the fitted waveform and the echo waveform, analyze the accuracy of the improved Gaussian decomposition algorithm, and provide a comparison for the subsequent analysis of the accuracy of the hardware structure.

(12) $$\mathrm{RMSE}=\sqrt{\frac{1}{n}\sum_{i=1}^{n}{\left(y_{i}-f\left(x\right)\right)}^{2}},$$

where $f\left(x\right)$ represents the value of the fitted waveform and $y_{i}$ represents the laser radar return waveform. These indicators reflect the overall accuracy of the fitted waveform between the fitted waveform and the original waveform. The standard of fitting is shown in Equation (13):

(13) $$\sigma_{T}\leq 3\sigma_{n}=\varepsilon ,$$

where $\sigma_{T}$ represents the residual between the original and filtered data, $\sigma_{n}$ represents the residual between the original and fitted data, and ε represents the triple residual.

The land echo waveform shown in Appendix A Figure A1a,b, were analyzed, where the FPGA run resulted in a double precision floating-point type.

The 64-bit double precision floating point type was converted to decimal, with two decimal places retained (see Table 1). Table 1 shows the decomposition results of Appendix A Figure A1a,b, Table 2 shows the result of MATLAB running and processing. The parameters include echo number, central location, pulse width, amplitude, and root mean square error (RMSE). The three parameters that make up the Gaussian component are the center position, pulse width, and amplitude. Each Gaussian component represents a different layer of the map. For example, each 2 ns corresponds to a one-way correction range of 0.2997 m. As shown in the results in Table 1, the different reflection areas (canopy) at these two footprint positions led to the following: (i) the average height detected in the first group was 44.47 m, 18.21 m, and the ground, and (ii) the average height detected in the second group was 26.54 m, 11.96 m, and the ground. The relative height of the ground for the first and second groups was 0.47 m.

Comparing the results in Table 2 and Table 3, the following was found:

(i) The deviation of the center position of the second Gaussian component in the first group of waveforms was 0.01, and the rest were the same;
(ii) The pulse width error was generally 0.01;
(iii) The amplitude error was relatively large, ranging from 0 to 0.16, but the amplitude value had no effect on the distance measurement point;
(iv) Half of the distance measurement point had an error of 0.01—that is, the error was 0.0029 m—and the ratio of the order of magnitude to the distance measurement of this project was 10^−2^. Combining the above data, the result of FPGA operation basically met the requirements.

**Figure 16:**
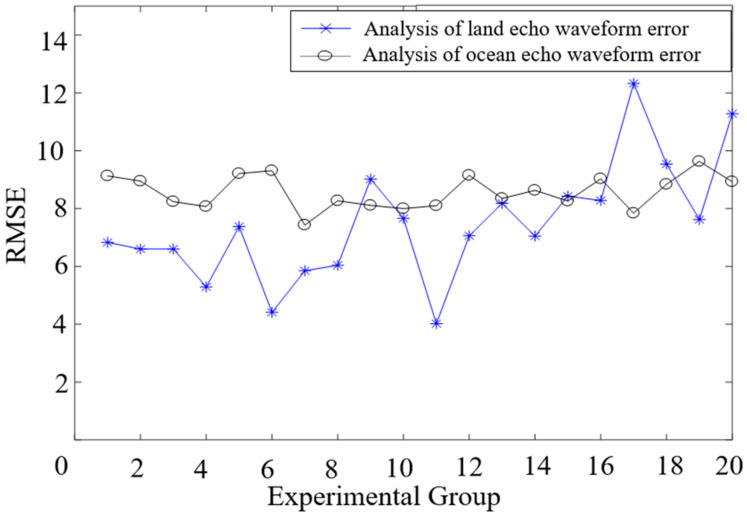
Error analysis of echo waveform.

The root mean square error was calculated for the forty sets of arithmetic results shown in Appendix A, Figure A1, and Appendix B, Figure A2, as shown in Figure 16. The blue line represents the error analysis for the land echo waveform, and the black line represents the error analysis of the ocean echo waveform. The experimental results basically met the requirements of error analysis.

The land echo waveform generally had more components and, so, the uncertainty of the error analysis was greater. Compared with the land echo waveform RMSE, the error of the ocean echo waveform was more stable. Although the land echo waveform was more complicated than the ocean echo waveform, the root mean square error of the land echo waveform in the whole was smaller than the root mean square error of the ocean echo waveform. Generally speaking, the fitting result still met the requirements.

### 4.4. Processing Speed and Hardware Consumption Resource Situation

The use of Vivado simulation waveforms allowed for analysis of the deviation of calculation results between the FPGA and the PC, the processing speed, and the consumption of hardware resources. The following were the main parameters of the PC: Windows 10 (64-bit), Intel(R) Core (TM) i5-7200U CPU@2 GHz with 4G RAM, and the MATLAB 2016b software. The selected chip was an XC7Z035FFG676-2 (Xilinx). The following were the main resources: 343,800 FFs, 11,900 LUTs, 500 Block Memory, and 900 DSPs. The price of PC and FPGA was about USD 700 and USD 400, respectively, and the power consumption was about 65 W and 30 W, respectively.

From the perspective of FPGA resource consumption analysis, LUTs were used the most in the hardware architecture of this paper, which was 7215; followed by 3389 FFs; 32 DSPs; 6.50 BRAMs; and 33 LUTRAM. In summary, the resource consumption (see Table 3) can be summarized as follows: LUTs used the most (which is 60%); FFs used 0.9%; Block RAMs used 7.9%; and DSPs used 3.5%.

The running time on the PC was 12 ms, while the simulation time on the FPGA was 0.041 ms; thus, the time acceleration ratio was 292. Therefore, when the LiDAR with laser emission frequency increased to 24 kHz, the algorithm was fully able to meet the real-time requirements on the FPGA.

## 5. Conclusions

In this paper, an improved Gaussian decomposition algorithm is proposed. The corresponding FPGA-based hardware architecture is implemented, which consists of six sub-modules: (1) pipeline data reading module, (2) Gaussian filtering module, (3) second-order difference module, (4) inflection point calculation module, (5) parameter solving module, and (6) output module. The functions of each sub-module are simulated through Vivado to verify the improved Gaussian decomposition algorithm using the experimental data. The decomposition errors of the echo signal waveform were analyzed.

Finally, a comparative analysis of the processing speed was conducted and the resource consumption of the hardware structure was validated. The experimental results show that the processing speed for the LiDAR full waveform decomposition using the proposed method is 292 times faster than that using a PC-based processing system without losing the accuracy of signal processing.

## Figures and Tables

**Table 1:** FPGA terrestrial LiDAR echo decomposition results.

Number	Echo Number	Central Location (Bins)	Pulse Width (Bins)	Amplitude (Counts)	RMSE
Figure A1a	3	546.74	24.27	206.19	6.83
		631.87	14.41	40.71	
		692.96	15.60	37.33	
Figure A1b	3	605.78	14.87	117.70	6.60
		655.82	7.10	38.72	
		693.81	12.64	43.41	

**Table 2:** Matlab terrestrial LiDAR echo decomposition results.

Number	Echo Number	Central Location (Bins)	Pulse Width (Bins)	Amplitude (Counts)	RMSE
Figure A1a	3	546.74	24.27	206.03	6.83
		631.88	14.40	40.71	
		692.96	15.59	37.34	
Figure A1b	3	605.78	14.86	117.63	6.60
		655.82	7.11	38.73	
		693.81	12.65	43.41	

**Table 3:** FPGA hardware resource consumption (xc7z035ffg676-2).

Resources	Consumption	Percentage of Total Resources
FFs	3389	0.9%
LUTs	7215	60%
Memory LUTs	39.5	7.9%
DSP48s	32	3.5%
